# Polyglutamine Ataxias in Denmark: Incidence and Relative Frequencies of SCA1, 2, 3, 6, 7, 17 and DRPLA in a Nationwide Cohort

**DOI:** 10.1007/s12311-025-01815-0

**Published:** 2025-03-14

**Authors:** Rosa Dam Waerling, Jenny Blechingberg, Jesper Kayser, Suzanne Granhøj Lindquist, Tua Vinther-Jensen, Jørgen Erik Nielsen, Morten Duno

**Affiliations:** 1 Department of Clinical Genetics, Rigshospitalet, Copenhagen University Hospital, Copenhagen, Denmark; 2 Neurogenetics Clinic and Research Lab, Danish Dementia Research Centre, Department of Neurology, Rigshospitalet, Copenhagen University Hospital, Copenhagen, Denmark; 3 Department of Clinical Genetics, Aarhus University Hospital, Aarhus, Denmark; 4 Department of Clinical Medicine, University of Copenhagen, Copenhagen, Denmark; 5 Department of Neurology, Bispebjerg Hospital, Copenhagen University Hospital, Copenhagen, Denmark

**Keywords:** Polyglutamine ataxia, Spinocerebellar ataxia, Dentatorubral-pallidoluysian atrophy, Incidence of SCA1, SCA2, SCA3, SCA6, SCA7, SCA17, DRPLA

## Abstract

Polyglutamine ataxias are autosomal dominantly inherited neurodegenerative disorders in which the molecular aetiology is an expanded CAG glutamine-encoding repeat in the causative genes. At present, there is no effective treatment, but there are several ongoing trials addressing polyglutamine disorders increasing the need of knowledge about prevalence and relative frequencies of the different subtypes. To identify all individuals with genetically confirmed polyglutamine ataxia in Denmark, determine frequency of subtypes and estimate the incidences. Retrospective data on all clinical tests performed on polyglutamine ataxias in Denmark during the last 15 years. 215 individuals with alleles in the full penetrant pathogenic range of polyglutamine ataxia genes were identified. The most frequent polyglutamine ataxia in Denmark was SCA6 which accounts for 42% of the identified individuals, followed by SCA2 and SCA3 which both account for approximately 20% each. Incidence rates were calculated. The study reveals the subtype distribution and incidence of polyglutamine ataxias in Denmark, priming the Danish cohort for future clinical trials as developments in the treatment of polyglutamine ataxia advances.

**Supplementary Information:**

The online version contains supplementary material available at 10.1007/s12311-025-01815-0.

## Introduction

Hereditary ataxias encompass a clinically and genetically heterogeneous group of disorders, where spinocerebellar ataxias (SCAs) is the most common subgroup, with more than 50 distinct clinical entities [1, 2]. The most prevalent SCAs are repeat-disorders characterized by a pathogenic CAG expansion in the protein coding regions of the affected genes, and hence referred to as polyglutamine (polyQ) ataxias, although several other expansions, often of a more complex nature, have been identified recently [3]. The most common polyglutamine ataxias encompass SCA1, 2, 3, 6, 7, 17 and dentatorubral-pallidoluysian atrophy (DRPLA) which are all autosomal dominantly inherited, but due to variation in age-of-onset and a heterogeneous clinical expression, an obvious family history is not always recognised.

The exact pathogenic mechanism of the CAG expansion is still largely unknown for the individual SCAs, but a toxic gain-of-function of the transcribed RNA per se and/or abnormal protein accumulation through polyQ aggregation mainly in the neurons of the cerebellum, has been suggested [4]. Several treatment regimens directed towards polyglutamine ataxias have been assessed over the years, but none have so far proven efficient in clinical practice [5]. However, antisense oligonucleotide (ASO) therapy has shown promising results [6], and clinical trials employing ASOs targeting SCA1 and SCA3 have been initiated [7, 8]. The advancement of future potential treatment regimens increases the need for knowledge concerning prevalence and distribution of polyglutamine ataxia in different population.

Both prevalence and the relative frequency of polyglutamine ataxias are highly variable across studied populations. SCA1 and SCA2 are reported to have comparable relative frequencies throughout Europe of about 25% whereas SCA3 generally is the most prevalent disorder. SCA7 and 17 are rare but more local founder populations have been reported [9, 10].

No studies have yet investigated the incidence and relative frequency of polyglutamine ataxias in Denmark. We here describe the entire SCA patient cohort identified in Denmark since testing of the different polyglutamine ataxias became available in a formalized nationwide clinical genetic setting in 2009.

## Methods

Retrospective data was collected from two public clinical genetic laboratories at Copenhagen University Hospital, Rigshospitalet (RH) and Aarhus University Hospital (AUH), Denmark, located in the Eastern and Western part of Denmark, respectively; the only two laboratories performing clinical analyses of polyglutamine ataxias in Denmark. Analyses were performed by routine methodologies. Primers and conditions are available upon reasonable request. Both sites are accredited according to ISO15189. Data was collected from 1317 individuals (symptomatic and presymptomatic) referred for spinocerebellar ataxia types 1, 2, 3, 6, 7, 17 and DRPLA testing (761 from RH and 556 from AUH). Prenatal analyses were excluded. Information about ethnicity was not available. Clinical testing for SCA1, 2, 3 and 6 has been performed since January 2009. Analysis for SCA17 and DRPLA was implemented in 2012, whereas analysis for SCA7 was added in 2014. The inclusion period thus covered 15 years for SCA1, 2, 3 and 6. All analyses were performed in a clinical setting after informed consent, according to best practice guidelines for SCA analysis [11]. Alleles were classified as normal, intermediate (if relevant) and pathogenic (supplementary data, Table 1), according to established ranges [11, 12]. Only alleles assigned to the pathogenic range where recorded.

**Table 1:** Number of individuals identified yearly with the different SCA subtypes

	2009	2010	2011	2012	2013	2014	2015	2016	2017	2018	2019	2020	2021	2022	2023	pr/year
SCA1	1			1	3	2	1		3		1	1	1	2	2	1,2
SCA2	−	7	4	8	3	2	3	1	2	5	4		3		2	2,9
SCA3	2			7	3	2	2	5	4	7	2	2	2	3	1	2,8
SCA6	5	2	2	8	4	12	11	5	5	4	11	5	1	8	7	6
SCA7						1										na
SCA17				2	2	1				1	2		2	2	2	1,2
DRPLA				1	1	1				2			1			0,5

Analyses for SCA17 and DRPLA was implemented in 2012, whereas analyses for SCA7 was implemented in 2014. Analyses for SCA 1, 2 3 and 6 has been performed since 2009. pr/year: average pick-up pr year (total nr. patient divided by number of years tested)

Data on birth cohorts was obtained from Statistics Denmark which is the central authority on Danish statistics (www.dst.dk). Incidence estimates were rounded to the nearest thousand.

According to Danish law, approval by the ethical review board is not required for the study, as no new data are generated, and the extracted data is anonymized. Legal approval for compiling historical data is granted by work zone nr: 22,045,498.

## Results

**Fig. 1:**
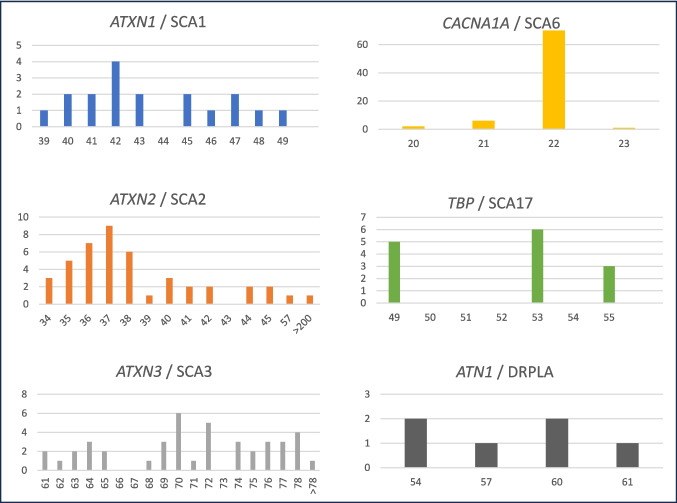
Distribution of the pathogenic alleles of the different SCA subtypes. X-axis depicts repeat-count, and y-axis the number of patients with the individual pathogenic repeat. SCA7 is omitted. The full dataset is provided in the supplementary data

During the inclusion period 1317 individuals were assessed for SCA 1, 2, 3, 6, 7, 17 and DRPLA, and were all included in the analysis. The majority were tested for multiple repeat expansions. Three individuals were heterozygous for an allele associated with reduced penetrance in *ATNX2* (SCA2: 34CAG), but all were symptomatic and included in the survey. A total of 215 individuals were identified with an abnormal expansion resulting in a diagnostic yield rate of 16%. All 215 were heterozygous for a single expansion. The size distribution of the expanded alleles is depicted in Fig. 1 (underlying data is listed in the supplementary data). The average yearly pick-up rate for the individual subtypes was calculated (Table 1).

The most frequent subtype was SCA6 accounting for 42% of the affected individuals, followed by SCA2 and SCA3 both constituting approximately 20%. The remaining subtypes are relatively rare in the Danish population (Table 2). The mean age at molecular diagnosis did not vary notably between the different subtypes although SCA6 patients tended to be slightly older. The gender distribution was as expected, except for SCA3 and SCA17 where males and females, respectively, were somewhat overrepresented, probably due to low numbers. The vast majority of affected individual were born between 1940 and 1990 where the mean yearly birth rate in Denmark was approx. 70.200, which was used to estimate the incidence of the different SCA subtypes in the Danish population (Table 2).

**Table 2:** Distribution of the different SCA subtypes and demographics

	Number (M/F)	RF^1^%	Mean age at diagnosis^2^	Male %	Incidence
SCA1	18 (10/8)	8,5	45 ± 18	55.5	1:70.000
SCA2	44 (19/25)	20	46 ± 17	43.2	1:25.000
SCA3	42 (28/14)	20	47 ± 15	66.6	1:25.000
SCA6	90 (47/43)	42	58 ± 15	52.2	1:12.000
SCA7	1 (0/1)	0,5	na	na	na
SCA17	14 (5/9)	6,5	44 ± 13	35.7	1:60.000
DRPLA	6 (2/4)	2,5	47 ± 13	na	1:140.000
Total	215				

1) Relative frequency. 2) Age at molecular diagnosis, ± standard deviation. *M/F* male/female, *Na* not applicable

## Discussion

Clinical testing for SCA1, 2, 3 and 6 has been available in Denmark since 2009, followed by SCA17 and DRPLA in 2012, and SCA7 in 2014. During this 15-year period (2009–2023) 1317 individuals were tested and of those 215 were diagnosed with one of the SCA subtypes, resulting in a diagnostic yield of 16%. The long observation period allows us to calculate the average number of individuals diagnosed pr. year within each subtype, except SCA7, and on the basis of the mean birth-rate relative to the number of diagnosed individuals within each subtype, estimate the incidence (Table 2). The number of diagnosed individuals were roughly distributed evenly over the test-period inferring data consistency, however the dataset did not allow for separating probands from family members, and thus, the incidence rate should be interpreted with caution. However, as all disorders are fully penetrant potential presymptomatic individuals will eventually develop symptoms and are thus not expected to bias the predicted incidence rates significantly.

Our analysis reveals SCA6 as the most frequent polyglutamine ataxia subtype in Denmark, accounting for 42% of diagnosed individuals, followed by SCA2 and SCA3, both accounting for 20%. This is in contrast to Central and Southern Europe where SCA3 is the most prevalent subtype [13–15] and where SCA1 and SCA2 often are found at comparable relative frequencies [9]. The identified SCA6 individuals were equally distributed between the two clinical sites located in different parts of Denmark (43 and 47, respectively), arguing against a founder effect, although a potential ancient founder cannot be excluded. The mean age of patients at molecular diagnosis for SCA6 tended to be slightly higher compared to other SCAs. It is well recognized that SCA6 has a relative late debut, but our observation could also reflect a latency from symptoms debut to molecular testing. This might also be true for the SCA1, SCA2 and SCA3 where age-at-diagnosis is somewhat higher than the generally reported age-at-onset. There could be several reasons for this apparent delay, but as the disorders are all progressive in nature, patients might not seek medical attention before symptoms affect daily functions. Delayed molecular confirmation could potential postpone or even prevent predictive testing, which could have clinical implications for family members.

SCA6 is also the most common subtypes in the North of England [16]. One could speculate that SCA6 may be common in North of England due to the influence of the Vikings, who might have enriched the subtype through journeys to England and intermarriage. Such spreading of alleles by activities of the Vikings has also been suggested for hereditary hemochromatosis [17] as well as Dupuytren’s disease [18]. Of note, SCA6 appears to be rare in the Norwegian population where SCA3 occurs as the most frequent subtype as in Central Europe [19]. In fact, there appears to be no consistency in the relative frequencies nor prevalence of the different SCAs, among the Scandinavian countries. SCA7 is relatively common in the Swedish and Finnish populations, due to a founder effect, whereas SCA7 is virtually absent from Norway and Denmark [10]. The single identified SCA7 individual in our series originates from Venezuela (personal communication) where SCA7 is the most prevalent SCA subtype [20]. Numerous founder effects explaining regional SCA frequencies have been described across different European populations [9].

### Limitations

Our data summarize all clinically referred molecular genetic testing of polyglutamine ataxia in the entire Danish population; however not all SCA subtypes were available for clinical testing during the entire 15 years of sampling. The incidence estimates, especially for SCA17 and DRPLA, are based on quite few individuals and should be interpreted with caution. The data did not allow for a complete separation of probands and relatives, and a subset of identified individuals might have been presymptomatic at the time of testing, but as the disorders show near complete penetrance, the incidence estimates should not be significantly affected.

## Conclusion

We have determined the relative frequency of polyglutamine ataxia subtypes in Denmark and estimated the incidence for common subtypes. In contrast to most other European populations, SCA6 is the most common subtype in Denmark whereas SCA7 is virtually absent from the indigenous Danish population. The study adds to population specific data on the frequency and incidence of polyglutamine ataxia subtypes, which is relevant for potential future clinical trials as new treatment options for SCA become available.

## Supplementary Information

Below is the link to the electronic supplementary material.

Supplementary file1 (DOCX 48 KB)

## Data Availability

No datasets were generated or analysed during the current study.
