# Supplementary material for: Polyglutamine Ataxias in Denmark: Incidence and Relative Frequencies of SCA1, 2, 3, 6, 7, 17 and DRPLA in a Nationwide Cohort
Source: Cerebellum. 2025 Mar 14;24(3):61. doi: 10.1007/s12311-025-01815-0 (PMC11909032; doi:10.1007/s12311-025-01815-0)
Supplement: Supplementary file 1 — Supplementary file1 (DOCX 48 KB) [file 12311_2025_1815_MOESM1_ESM.docx]

**Supplementary data**

**Supplementary table 1.**

Reference intervals for the different CAG repeats.

| Gene (disorder) | Normal | Intermediate^1^ | Pathogenic^2^ |
| --- | --- | --- | --- |
| *ATXN1* (SCA1) | <36^3^ | 36-38^4^ | >38 |
| *ATXN2* (SCA2) | <32^5^ | 32-34 | >34 |
| *ATXN3* (SCA3) | <45 | 45-60^6^ | >60 |
| *CACNA1A* (SCA6) | <19 | 19 | >19 |
| *ATXN7* (SCA7) | <28 | 28-36 | >36 |
| *TBP* (SCA17) | <41 | 41-48 | >48 |
| *ATN1* (DRPLA) | <36 | 36-47 | >47 |

1) Intermediate and/or reduced penetrance / questionable / unknown

2) Full penetrant. The error margin is up to +/- 2 units. Patients with expansions crossing pathogenic cut-off, are included if the phenotype was highly consistent with the specific disorder

3) Up to 44 if interrupted by CAT

4) Without CAT interruptions

5) 31 repeat allele is associated with recessive SCA2

6) The exact cut-off between intermediate and pathogenic is not well defined

The ranges are adapted from GeneReviews, <https://www.ncbi.nlm.nih.gov/books/NBK1116/>

**Distribution of pathogenic alleles pr. gene**

***ATXN1* / SCA1**

| Rep | Allele 1 | Allele 2 | Gender | Age at test |
| --- | --- | --- | --- | --- |
| ATXN1 | 30 CAG | 40 CAG | Female | 60 |
| ATXN1 | 30 CAG | 40 CAG | Female | 74 |
| ATXN1 | 29 CAG | 41 CAG | Female | 25 |
| ATXN1 | 33 CAG | 42 CAG | Female | 56 |
| ATXN1 | 29 CAG | 42 CAG | Male | 54 |
| ATXN1 | 29 CAG | 42 CAG | Male | 57 |
| ATXN1 | 32 CAG | 42 CAG | Male | 55 |
| ATXN1 | 29 CAG | 45 CAG | Male | 36 |
| ATXN1 | 32 CAG | 45 CAG | Female | 28 |
| ATXN1 | 32 CAG | 46 CAG | Male | 45 |
| ATXN1 | 31 CAG | 49 CAG | Female | 55 |
| ATXN1 | 30 CAG | 39 CAG | Male | 11 |
| ATXN1 | 30 CAG | 47 CAG | Female | 49 |
| ATXN1 | 29 CAG | 41 CAG | Male | 70 |
| ATXN1 | 36 CAG | 47 CAG | Male | 41 |
| ATXN1 | 30 CAG | 43 CAG | Male | 17 |
| ATXN1 | 33 CAG | 48 CAG | Female | 27 |
| ATXN1 | 31 CAG | 43 CAG | Male | 50 |

***ATXN2* / SCA2**

| Rep | Allele 1 | Allele 2 | Gender | Age at test |
| --- | --- | --- | --- | --- |
| ATXN2 | 22 CAG | >200 CAG | Female | 15 |
| ATXN2 | 22 CAG | 45 CAG | Male | 49 |
| ATXN2 | 22 CAG | 35 CAG | Male | 56 |
| ATXN2 | 27 CAG | 41 CAG | Female | 35 |
| ATXN2 | 22 CAG | 36 CAG | Male | 56 |
| ATXN2 | 22 CAG | 36 CAG | Female | 20 |
| ATXN2 | 22 CAG | 36 CAG | Female | 24 |
| ATXN2 | 22 CAG | 40 CAG | Female | 77 |
| ATXN2 | 22 CAG | 38 CAG | Female | 48 |
| ATXN2 | 22 CAG | 41 CAG | Male | 55 |
| ATXN2 | 22 CAG | 42 CAG | Male | 32 |
| ATXN2 | 22 CAG | 44 CAG | Female | 25 |
| ATXN2 | 22 CAG | 36 CAG | Female | 68 |
| ATXN2 | 22 CAG | 36 CAG | Male | 57 |
| ATXN2 | 22 CAG | 44 CAG | Female | 57 |
| ATXN2 | 22 CAG | 35 CAG | Female | 66 |
| ATXN2 | 22 CAG | 37 CAG | Female | 45 |
| ATXN2 | 22 CAG | 35 CAG | Male | 56 |
| ATXN2 | 22 CAG | 37 CAG | Female | 40 |
| ATXN2 | 22 CAG | 45 CAG | Male | 51 |
| ATXN2 | 22 CAG | 42 CAG | Male | 25 |
| ATXN2 | 22 CAG | 37 CAG | Female | 24 |
| ATXN2 | 22 CAG | 57 CAG | Female | 18 |
| ATXN2 | 22 CAG | 35 CAG | Male | 68 |
| ATXN2 | 22 CAG | 34 CAG | Male | 50 |
| ATXN2 | 22 CAG | 35 CAG | Male | 72 |
| ATXN2 | 22 CAG | 38 CAG | Female | 39 |
| ATXN2 | 22 CAG | 36 CAG | Male | 54 |
| ATXN2 | 22 CAG | 40 CAG | Male | 22 |
| ATXN2 | 22 CAG | 38 CAG | Female | 59 |
| ATXN2 | 22 CAG | 38 CAG | Male | 61 |
| ATXN2 | 22 CAG | 38 CAG | Female | 52 |
| ATXN2 | 22 CAG | 37 CAG | Male | 64 |
| ATXN2 | 22 CAG | 37 CAG | Female | 77 |
| ATXN2 | <31 CAG | 39 CAG | Female | 48 |
| ATXN2 | 22 CAG | 34 CAG | Female | 48 |
| ATXN2 | 22 CAG | 37 CAG | Female | 23 |
| ATXN2 | <31 CAG | 34 CAG | Female | 23 |
| ATXN2 | 22 CAG | 37 CAG | Female | 37 |
| ATXN2 | 22 CAG | 40 CAG | Male | 24 |
| ATXN2 | 22 CAG | 37 CAG | Female | 50 |
| ATXN2 | 22 CAG | 37 CAG | Female | 64 |
| ATXN2 | 22 CAG | 36 CAG | Male | 70 |
| ATXN2 | 22 CAG | 38 CAG | Male | 32 |

**ATXN3 / SCA3**

| Rep | Allel 1 | Allele 2 | Gender | Age at test |
| --- | --- | --- | --- | --- |
| ATXN3 | 22 CAG | 70 CAG | Male | 45 |
| ATXN3 | 14 CAG | 77 CAG | Male | 48 |
| ATXN3 | 28 CAG | 75 CAG | Male | 47 |
| ATXN3 | 14 CAG | 78 CAG | Male | 58 |
| ATXN3 | 38 CAG | 75 CAG | Female | 43 |
| ATXN3 | 24 CAG | 82 CAG | Male | 25 |
| ATXN3 | 23 CAG | 74 CAG | Male | 42 |
| ATXN3 | 14 CAG | 77 CAG | Male | 46 |
| ATXN3 | 30 CAG | 76 CAG | Female | 36 |
| ATXN3 | 14 CAG | 76 CAG | Male | 64 |
| ATXN3 | 27 CAG | 78 CAG | Male | 33 |
| ATXN3 | 16 CAG | 76 CAG | Female | 31 |
| ATXN3 | 16 CAG | 63 CAG | Male | 61 |
| ATXN3 | 16 CAG | 72 CAG | Male | 24 |
| ATXN3 | 25 CAG | 72 CAG | Male | 36 |
| ATXN3 | 16 CAG | 74 CAG | Female | 31 |
| ATXN3 | 25 CAG | 74 CAG | Female | 62 |
| ATXN3 | 22 CAG | 72 CAG | Male | 50 |
| ATXN3 | 32 CAG | 78 CAG | Female | 19 |
| ATXN3 | 25 CAG | 65 CAG | Female | 60 |
| ATXN3 | 15 CAG | 77 CAG | Male | 52 |
| ATXN3 | 25 CAG | 72 CAG | Male | 30 |
| ATXN3 | 23 CAG | 78 CAG | Female | 22 |
| ATXN3 | 14 CAG | 68 CAG | Male | 26 |
| ATXN3 | 27 CAG | 61 CAG | Female | 30 |
| ATXN3 | 14 CAG | 63 CAG | Male | 78 |
| ATXN3 | 14 CAG | 69 CAG | Male | 54 |
| ATXN3 | 14 CAG | 62 CAG | Male | 62 |
| ATXN3 | 14 CAG | 69 CAG | Male | 30 |
| ATXN3 | 24 CAG | 71 CAG | Male | 46 |
| ATXN3 | 24 CAG | 64 CAG | Male | 73 |
| ATXN3 | 24 CAG | 64 CAG | Female | 61 |
| ATXN3 | 23 CAG | 65 CAG | Male | 65 |
| ATXN3 | 23 CAG | 70 CAG | Male | 52 |
| ATXN3 | 14 CAG | 72 CAG | Female | 60 |
| ATXN3 | 28 CAG | 70 CAG | Male | 51 |
| ATXN3 | 21 CAG | 69 CAG | Female | 68 |
| ATXN3 | 13 CAG | 70 CAG | Male | 61 |
| ATXN3 | 14 CAG | 70 CAG | Female | 48 |
| ATXN3 | 14 CAG | 70 CAG | Male | 44 |
| ATXN3 | 19 CAG | 64 CAG | Female | 22 |
| ATXN3 | 16 CAG | 61 CAG | Male | 72 |

**CACNA1A / SCA6**

| Rep | Allele 1 | Allele 2 | Gender | Age at test |
| --- | --- | --- | --- | --- |
| CACNA1A | 14 CAG | 22 CAG | Female | 78 |
| CACNA1A | 12 CAG | 22 CAG | Female | 45 |
| CACNA1A | 11 CAG | 22 CAG | Male | 61 |
| CACNA1A | 7 CAG | 22 CAG | Male | 51 |
| CACNA1A | 11 CAG | 22 CAG | Male | 68 |
| CACNA1A | 13 CAG | 22 CAG | Female | 65 |
| CACNA1A | 12 CAG | 22 CAG | Female | 39 |
| CACNA1A | 14 CAG | 21 CAG | Female | 58 |
| CACNA1A | 7 CAG | 22 CAG | Female | 39 |
| CACNA1A | 13 CAG | 22 CAG | Male | 60 |
| CACNA1A | 11 CAG | 22 CAG | Male | 76 |
| CACNA1A | 12 CAG | 22 CAG | Female | 74 |
| CACNA1A | 11 CAG | 22 CAG | Female | 69 |
| CACNA1A | 13 CAG | 22 CAG | Male | 66 |
| CACNA1A | 13 CAG | 23 CAG | Male | 62 |
| CACNA1A | 11 CAG | 22 CAG | Male | 67 |
| CACNA1A | 11CAG | 22 CAG | Female | 59 |
| CACNA1A | 12 CAG | 22 CAG | Male | 65 |
| CACNA1A | 13 CAG | 22 CAG | Female | 66 |
| CACNA1A | 7 CAG | 22 CAG | Male | 56 |
| CACNA1A | 13 CAG | 22 CAG | Male | 62 |
| CACNA1A | 11 CAG | 20 CAG | Male | 82 |
| CACNA1A | 11 CAG | 22 CAG | Male | 72 |
| CACNA1A | 11 CAG | 22 CAG | Female | 79 |
| CACNA1A | 13 CAG | 22 CAG | Male | 54 |
| CACNA1A | 11 CAG | 22 CAG | Male | 65 |
| CACNA1A | 7 CAG | 22 CAG | Female | 68 |
| CACNA1A | 13 CAG | 22 CAG | Female | 62 |
| CACNA1A | 11 CAG | 22 CAG | Male | 23 |
| CACNA1A | 13 CAG | 22 CAG | Female | 56 |
| CACNA1A | 13 CAG | 22 CAG | Female | 42 |
| CACNA1A | 11 CAG | 22 CAG | Female | 30 |
| CACNA1A | 14 CAG | 22 CAG | Female | 37 |
| CACNA1A | 13 CAG | 22 CAG | Female | 70 |
| CACNA1A | 11 CAG | 22 CAG | Female | 60 |
| CACNA1A | 11 CAG | 22 CAG | Male | 60 |
| CACNA1A | 11 CAG | 22 CAG | Male | 75 |
| CACNA1A | 11 CAG | 22 CAG | Male | 33 |
| CACNA1A | 13 CAG | 22 CAG | Male | 65 |
| CACNA1A | 7 CAG | 20 CAG | Male | 58 |
| CACNA1A | 13 CAG | 22 CAG | Male | 75 |
| CACNA1A | 13 CAG | 22 CAG | Female | 44 |
| CACNA1A | 11 CAG | 22 CAG | Female | 37 |
| CACNA1A | 11 CAG | 22 CAG | Male | 55 |
| CACNA1A | 12 CAG | 22 CAG | Female | 70 |
| CACNA1A | 12 CAG | 22 CAG | Female | 52 |
| CACNA1A | 13 CAG | 22 CAG | Male | 76 |
| CACNA1A | 5 CAG | 22 CAG | Female | 25 |
| CACNA1A | 13 CAG | 22 CAG | Female | 43 |
| CACNA1A | 11 CAG | 22 CAG | Female | 77 |
| CACNA1A | 11 CAG | 22 CAG | Male | 63 |
| CACNA1A | 13 CAG | 22 CAG | Male | 71 |
| CACNA1A | 14 CAG | 22 CAG | Male | 38 |
| CACNA1A | 8 CAG | 22 CAG | Female | 32 |
| CACNA1A | 14 CAG | 22 CAG | Male | 65 |
| CACNA1A | 13 CAG | 22 CAG | Male | 62 |
| CACNA1A | 11 CAG | 22 CAG | Female | 84 |
| CACNA1A | 13 CAG | 22 CAG | Male | 47 |
| CACNA1A | 13 CAG | 22 CAG | Female | 40 |
| CACNA1A | 13 CAG | 22 CAG | Female | 62 |
| CACNA1A | 13 CAG | 22 CAG | Female | 35 |
| CACNA1A | 13 CAG | 22 CAG | Female | 63 |
| CACNA1A | 14 CAG | 22 CAG | Male | 35 |
| CACNA1A | 13 CAG | 22 CAG | Male | 46 |
| CACNA1A | 11 CAG | 22 CAG | Male | 66 |
| CACNA1A | 13 CAG | 22 CAG | Female | 71 |
| CACNA1A | 11 CAG | 22 CAG | Female | 45 |
| CACNA1A | 11 CAG | 22 CAG | Female | 72 |
| CACNA1A | 11 CAG | 22 CAG | Female | 63 |
| CACNA1A | 11 CAG | 22 CAG | Male | 82 |
| CACNA1A | 11 CAG | 22 CAG | Female | 63 |
| CACNA1A | 11 CAG | 22 CAG | Male | 48 |
| CACNA1A | 11 CAG | 22 CAG | Male | 31 |
| CACNA1A | 12 CAG | 21 CAG | Male | 66 |
| CACNA1A | 10 CAG | 21 CAG | Male | 61 |
| CACNA1A | 12 CAG | 22 CAG | Female | 75 |
| CACNA1A | 11 CAG | 22 CAG | Female | 65 |
| CACNA1A | 11 CAG | 21 CAG | Male | 32 |
| CACNA1A | 11 CAG | 21 CAG | Male | 67 |
| CACNA1A | 10 CAG | 22 CAG | Female | 65 |
| CACNA1A | 11 CAG | 22 CAG | Female | 71 |
| CACNA1A | 12 CAG | 22 CAG | Male | 67 |
| CACNA1A | <18 CAG | 22 CAG | Female | 30 |
| CACNA1A | 10 CAG | 22 CAG | Male | 62 |
| CACNA1A | 10 CAG | 22 CAG | Male | 62 |
| CACNA1A | 11 CAG | 22 CAG | Male | 63 |
| CACNA1A | 10 CAG | 21 CAG | Male | 52 |
| CACNA1A | <18 CAG | 22 CAG | Female | 69 |
| CACNA1A | 12 CAG | 22 CAG | Female | 49 |
| CACNA1A | 12 CAG | 22 CAG | Female | 39 |

**ATXN7 / SCA7**

| Rep | Allele 1 | Allele 2 | Gender | Age at test |
| --- | --- | --- | --- | --- |
| ATXN7 | 38 CAG | 10 CAG | Female | 63 |

**TBP / SCA17**

| Rep | Allele 1^1^ | Allele 2^1,2^ | Gender | Age at test |
| --- | --- | --- | --- | --- |
| TBP | 38 CAG/CAA | 49 CAG/CAA | Male | 50 |
| TBP | 39 CAG/CAA | 49 CAG/CAA | Female | 44 |
| TBP | 39 CAG/CAA | 53 CAG/CAA | Female | 44 |
| TBP | 38 CAG/CAA | 53 CAG/CAA | Male | 67 |
| TBP | 38 CAG/CAA | 53 CAG/CAA | Male | 33 |
| TBP | 39 CAG/CAA | 53 CAG/CAA | Female | 49 |
| TBP | 38 CAG/CAA | 49 CAG/CAA | Male | 58 |
| TBP | 37 CAG/CAA | 49 CAG/CAA | Female | 35 |
| TBP | 40 CAG/CAA | 53 CAG/CAA | Female | 19 |
| TBP | 38 CAG/CAA | 49 CAG/CAA | Female | 62 |
| TBP | 38 CAG/CAA | 53 CAG/CAA | Female | 51 |
| TBP | 33 CAG/CAA | 55 CAG/CAA | Female | 27 |
| TBP | 36 CAG/CAA | 55 CAG/CAA | Male | 38 |
| TBP | 38 CAG/CAA | 55 CAG/CAA | Female | 47 |

1) The exact repeat structure has not been determined. 2) The error margin is 2 +/- units. Patients with expansion crossing pathogenic cut-off, are included if phenotype was consistent with SCA17, and listed as 49 rep.

**ATN1 / DRPLA**

| Rep | Allele 1 | Allele 2 | Gender | Age at test |
| --- | --- | --- | --- | --- |
| ATN1 | 15 CAG | 54 CAG | Female | 65 |
| ATN1 | 10 CAG | 57 CAG | Female | 47 |
| ATN1 | 15 CAG | 54 CAG | Female | 59 |
| ATN1 | 16 CAG | 61 CAG | Male | 31 |
| ATN1 | 16 CAG | 60 CAG | Male | 33 |
| ATN1 | 14 CAG | 60 CAG | Female | 49 |
